# Protocol for analyzing antibody responses to glycoprotein antigens using electron-microscopy-based polyclonal epitope mapping

**DOI:** 10.1016/j.xpro.2023.102476

**Published:** 2023-07-28

**Authors:** Hannah L. Turner, Abigail M. Jackson, Sara T. Richey, Leigh M. Sewall, Aleksandar Antanasijevic, Lars Hangartner, Andrew B. Ward

**Affiliations:** 1 Department of Integrative Structural and Computational Biology, The Scripps Research Institute, La Jolla, CA 92037, USA; 2 Department of Immunology and Microbiology, The Scripps Research Institute, La Jolla, CA 92037, USA

**Keywords:** Immunology, Microscopy, Antibody, Structural Biology

## Abstract

Electron microscopy-based polyclonal epitope mapping (EMPEM) can delineate epitope specificities of serum antibodies to a given antigen following vaccination or infection. Here, we present a protocol for the EMPEM method for rapid high-throughput assessment of antibody responses to glycoprotein antigens in vaccination and infection studies. We describe steps for antibody isolation and digestion, antigen complex and purification, and electron microscope imaging. We then detail procedures for processing and analysis of EMPEM data.

For complete details on the use and execution of this protocol, please refer to Bianchi et al. (2018).^1^

## Highlights

• Electron-microscopy-based technique to visualize antibody-mediated immune response
• Quickly and efficiently visualize polyclonal antibody response to antigens
• Multi-species antibody isolation from clinical and pre-clinical samples
• Rapid high-throughput readout of on- and off-target responses to antigens

## Abstract (editor highlights)

Publisher’s note: Undertaking any experimental protocol requires adherence to local institutional guidelines for laboratory safety and ethics.

## Graphical abstract

**Figure:**
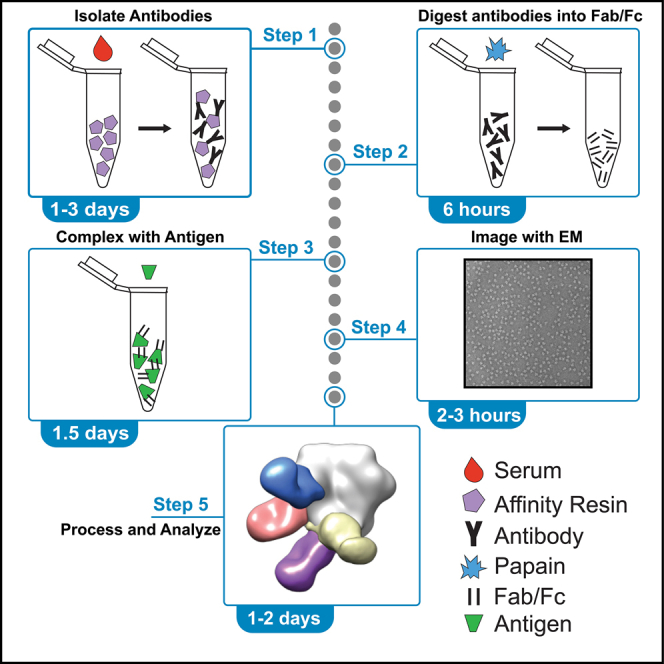

## Teaser

Electron-microscopy-based polyclonal epitope mapping (EMPEM) can delineate epitope specificities of serum antibodies to a given antigen following vaccination or infection. Here, we present a protocol for EMPEM protocol for rapid high-throughput assessment of antibody responses to glycoprotein antigens in vaccination and infection studies. We describe steps for antibody isolation and digestion, antigen complex and purification, and electron microscope imaging. We then detail procedures for processing and analysis of EMPEM data.

## Before you begin

The protocol below describes: isolation of human IgG from serum using Protein G, elution from resin and digestion with papain, addition of soluble HIV Env, complex purification, and deposition onto negative stain electron microscopy grids. However, this protocol can also be used to isolate IgG from animal models such as rabbit and non-human primates (NHP), with alternatives such as Protein A or CaptureSelect IgG-Fc resin, and other antigens of interest.

### Heat inactivation

**Timing: 1.5 h**

1. Heat inactivation of NHP or human serum/plasma.
  a. Thaw samples from −80°C to 20°C.
  b. Transfer samples to 56°C for 1 h.
  c. Place in biosafety cabinet (BSC) before starting protocol.

## Key resources table

**Table:** 

REAGENT or RESOURCE	SOURCE	IDENTIFIER
**Biological samples**		

Naïve human serum	Sigma-Aldrich	Cat #H4522-100mL

**Chemicals, peptides, and recombinant proteins**		

Papain from papaya latex	Sigma	CAS 9001-73-4
Tris base	VWR	CAS 77-86-1
EDTA tetrasodium salt	AMRESCO	CAS 13235-36-4
L-cysteine	Sigma-Aldrich	CAS 52-90-4
Iodoacetamide	Sigma-Aldrich	CAS 144-48-9
Uranyl formate hydrate	Electron Microscopy Sciences	Cat # 22541
Protein G resin sepharose 4 Fast Flow	Cytiva	17061805
rProtein A resin sepharose Fast Flow	Cytiva	17127903
Capture Select IgG-Fc affinity matrix	Thermo Scientific	191285550
Glycine	VWR	56-40-6
NaCl	Honeywell	Cat #S9888-10KG
NaOH	Fisher Scientific	UN1823
Sodium azide	TCI America	CAS 26628-22-8

**Software and algorithms**		

EPU	Thermo Scientific	Part # 1025080
Relion 3.0	Zivanov et al.^2^	https://www3.mrc-lmb.cam.ac.uk/relion/index.php/Main_Page
UCSF Chimera	Pettersen et al.^3^	Cgl.ucsf.edu/chimera/
Leginon	Suloway et al.^4^	PMID: 15890530
Appion	Lander et al.^5^	https://emg.nysbc.org/redmine/projects/appion
DoG Picker	Voss et al.^6^	https://emg.nysbc.org/redmine/projects/appion

**Other**		

AKTA Pure 25 M1	GE, Cytiva	N/A
Superdex 200 increase 10/300	Cytiva	289990944
Talos F200C TEM	Thermo Scientific	N/A
CETA 16M camera 200 kV	Thermo Scientific	N/A
Nanodrop 2000	Thermo Scientific	Cat # ND2000
Electron microscopy copper mesh grids	Electron Microscopy Sciences	Cat # EMS400-Cu
Pelco easiGlow Glow Discharge Cleaning System	Ted Pella Inc.	921000
0.22 μm pore filter	Cytiva	6780-2502
30 kDa 15 mL concentrator	Milipore	REF UFC803096
Sterile collodion 2% amyl acetate	Electron Microscopy Sciences	Cat # 12620-50
Graphite Rods	Ted Pella Inc.	Cat # 61-35
Cressington Carbon evaporator	Ted Pella Inc.	Model # 9620
Parafilm “M”	Bemis	PM-996
Filter paper 1	Whatman	Cat # 1001 090

## Materials and equipment

**Table:** 10× stock of Tris/EDTA pH 8

Reagent	Final concentration	Amount
Tris	1 M	6.06 g (MW 121.14 g/mol)
EDTA	20 mM	0.292 g (MW 292.24 g/mol)
ddH_2_O	N/A	Bring total volume up to 50 mL
**Total**	**N/A**	**50 mL**

Storage conditions: room temperature, up to 2 weeks.

**Table:** Activated Papain (40 μL per 1 mg of IgG)

Reagent	Final concentration	Amount
Papain	1% (w/w)	38.5 μL (at 26 mg/mL)
10× Tris/EDTA	1×	100 μL
10× L-cysteine	1×	100 μL
ddH_2_O	N/A	761.5 μL
**Total**	**N/A**	**1 mL**

***Note:*** Incubate for 15 min at 37°C to activate papain.

Storage conditions: Freshly prepared before use.

**Table:** 1× PBS pH 7.5

Reagent	Final concentration	Amount
NaCl	137 mM	8 g (MW 58.44 g/mol)
KCl	2.7 mM	0.202 g (MW 74.55 g/mol)
Na_2_HPO_4_	10 mM	1.42 g (MW 141.96 g/mol)
KH_2_PO_4_	1.8 mM	0.245 g (MW 136.09 g/mol)
ddH_2_O	N/A	Bring total volume up to 1 L
**Total**	**N/A**	**1 L**

• 10× L-cysteine solution: add 0.121 g L-cysteine (MW 121.16 g/mol) in 10 mL ddH_2_O.
  ○ Storage conditions: make fresh before use.
• 500 mM Iodoacetamide solution: add 4.634 g (MW 184.96 g/mol) in 50 mL ddH_2_O.
  ○ Storage conditions: aliquot and store indefinitely at −20°C.
• 0.1 M Glycine pH 2.5 solution: 3.75 g (MW 75.07 g/mol) in 500 mL.
  ○ Store indefinitely.
• 1 M Tris pH 8: 60.57 g (MW 121.14 g/mol) in 500 μL.
  ○ Store indefinitely.

**CRITICAL:** Uranyl formate (UF) is radioactive. Take caution when working with powder-form UF. Handle in chemical hood with mask and goggles.

• 2% Uranyl formate (MW 378.08 g/mol).
  ○ 0.1 g of powder-form uranyl formate in 5 mL ddH_2_O to achieve 2% UF.
    - Combine powder and ddH_2_O in opaque tube and vortex for 10 min.
    - Add 2 μL 10 N KOH per milliliter of ddH_2_O.
    - Vortex for 10 min then centrifuge for 10 min.
    - In a dimly lit room, filter solution with 0.22 μm syringe filter.
    - Aliquot 50 μL and place tubes in liquid nitrogen.
    - Transfer frozen tubes to −80°C.
  ○ Storage conditions: aliquot and store indefinitely in −80°C, light-sensitive.

## Step-by-step method details

### Isolate IgG from serum/plasma

**Timing: Buffer exchange 30 min**

**Timing: Incubation 1–3 days**

**Timing: Elution30 min**

This section uses affinity resin targeting the Fc region of an antibody to separate polyclonal IgG from serum or plasma.

1. Buffer exchange Protein G into 1× PBS pH 7.5.
  a. Aliquot 1 mL of resin (or 2 mL 50% slurry) per 1 mL serum/plasma into 15 mL conical.
  b. Centrifuge for 3 min at 3,200 × *g* and decant supernatant.
  c. Fill 15 mL conical with PBS, mix into resin, spin down, and decant.
  d. Repeat 2 more times.

***Alternatives:*** Swap Protein G with affinity resin better suited for a specific species (Table 1).

2. Resuspend resin with PBS back to original slurry volume.
  a. In BSC with proper personal protective equipment (PPE), combine 2 mL slurry and 1 mL serum in 15 mL conical.
  b. Bring volume up to 5 mL with PBS.
  c. Secure tube lid with parafilm.

**Table 1:** Affinity resins

Resin	Binding capacity	Strong binding to animal species (IgG)	Medium binding (IgG)
Protein G Sepharose 4 Fast Flow	17–23 mg IgG/mL	Human (all subtypes), NHP, rabbit, cow, horse	Rat, mouse, guinea pig, goat, sheep
rProtein A sepherose Fast Flow	35 mg IgG/mL	Human (not IgG_3_ subtype), NHP, rabbit, guinea pig, mouse (IgG_2_)	Cow, dog, horse, mouse (IgG_1_ and IgG_3_)
CaptureSelect IgG-Fc multispecies	25 mg IgG/mL	Human (all subtypes), NHP, mouse, rabbit, donkey, horse, hamster, sheep, guinea pig	Cow, goat, pig, cat, rat
3. Mix serum and resin for 1–3 days at 4°C.
4. Separate resin-bound IgG from serum.
  a. In BSC with proper PPE, remove parafilm and centrifuge for 3 min at 3,200 × *g*.
  b. Decant serum into 10% bleach.
  c. Fill 15 mL tube with PBS, mix and centrifuge for 3 min at 3,200 × *g*, decant into 10% bleach.
  d. Repeat 2 more times.
  e. Finish by adding 1 mL PBS to resin-bound IgG.

***Note:*** Sample is now safe to remove from BSC.

5. Elute IgG from Protein G resin.
  a. Add 5 mL (5 CV) 0.1 M Glycine pH 2.5 to resin-bound IgG slurry.
  b. Secure lid and incubate for 15 min at 20°C, gently mixing on rocker.
  c. Centrifuge for 3 min at 3,200 × *g*.
  d. Decant supernatant (IgG-containing) into 4 mL (4 CV) of 1 M Tris pH 8.

**Figure 1:**
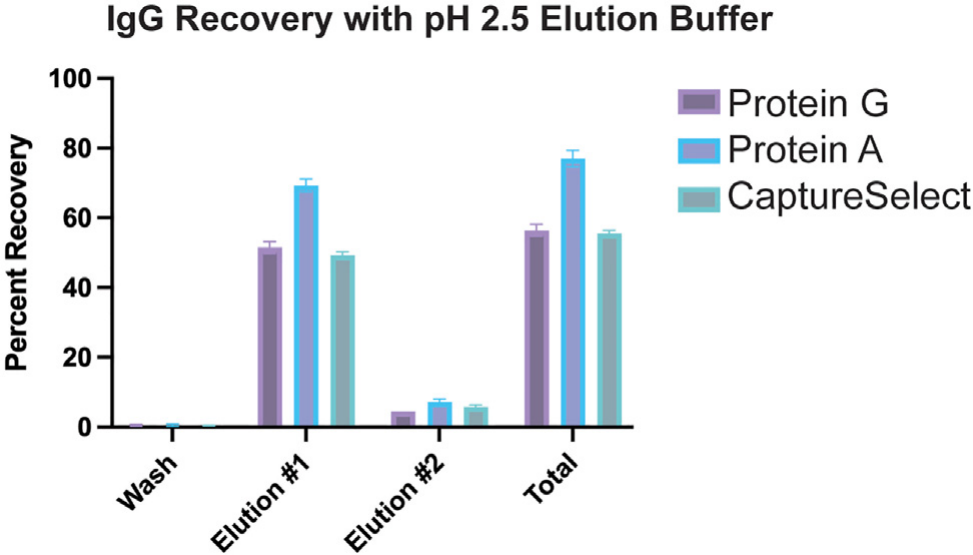
IgG Elution 2 mg of purified naïve human IgG was added to Protein G and CaptureSelect. 2 mg of purified NHP was added to protein A and incubated for 72 h at 4C. Total IgG recovery was recorded after 1 or 2 rounds of elution.
7. Buffer exchange into PBS with 30 or 100 kDa molecular cut-off 15 mL concentrator.
  a. Spin down IgG-containing elution buffer.
  b. Fill concentrator with PBS.
  c. Spin down to 0.5–1 mL total volume.
  d. Transfer IgG to microcentrifuge tube.
8. Measure IgG concentration (extinction coefficient 13.7 A1% 280 nm) and calculate total IgG.

***Note:*** IgG yield will vary depending on species and serum processing, among other things. From experience and recent studies, 5–10 mg of IgG is expected from 1 mL human or NHP serum, 3–8 mg from ferrets, and 1–5 mg from mice.

***Optional:*** Can perform another round of elution from resin (step 5) to recover maximum amount of IgG (Figure 1).

6. Filter elution into new tube using 0.2 μm (or 0.45 μm) pore filter to remove remaining resin beads.

**Pause point:** IgG can be stored at 4°C for up to 2 weeks, after which it is recommended to store at −80°C indefinitely.

### Digest IgG

**Timing: 6 h**

Papain enzyme is used to digest IgG into Fab and Fc fragments. It targets the hinge region of the antibody where the Fab domains connect to the Fc domain. We are interested in the paratope/epitope interaction of Fab and antigen. Complexes made with IgG will most likely cause aggregation and will be difficult to process. Troubleshooting 1.

***Note:*** Protocol calculated for 5 mg of IgG. Adjust ratios accordingly.

9. Activate papain.
  a. See recipe in materials and equipment.
  b. 200 μL of activated papain is required for 5 mg of IgG.
10. Combine 10× Tris/EDTA, 10× L-cysteine and isolated IgG with PBS for a total of 9.8 mL (total reaction of 10 mL) (Table 2).
  a. Add 200 μL activated papain.

**Table 2:** Digestion buffer

Component	Calculated for 1 mg IgG	Calculated for 5 mg IgG
IgG	1 mg	5 mg
Activated Papain	40 μL	200 μL
10× Tris/EDTA	200 μL	1 mL
10× L-cysteine	200 μL	1 mL
ddH_2_O	Bring total volume up to 2 mL	Bring total volume up to 10 mL
Total Reaction Volume	2 mL	10 mL
11. Incubate digestion reaction for 4–5 h at 37°C.
12. Quench reaction by adding 600 μL of 500 mM iodoacetamide (final concentration 30 mM).

**Pause point:** Digested Fab can be stored at 4°C for up to one month when 0.025% azide is added to prevent contamination growth. Place in −80°C for long-term storage.

### Fab/Fc purification

**Timing: 2 h**

**Figure 2:**
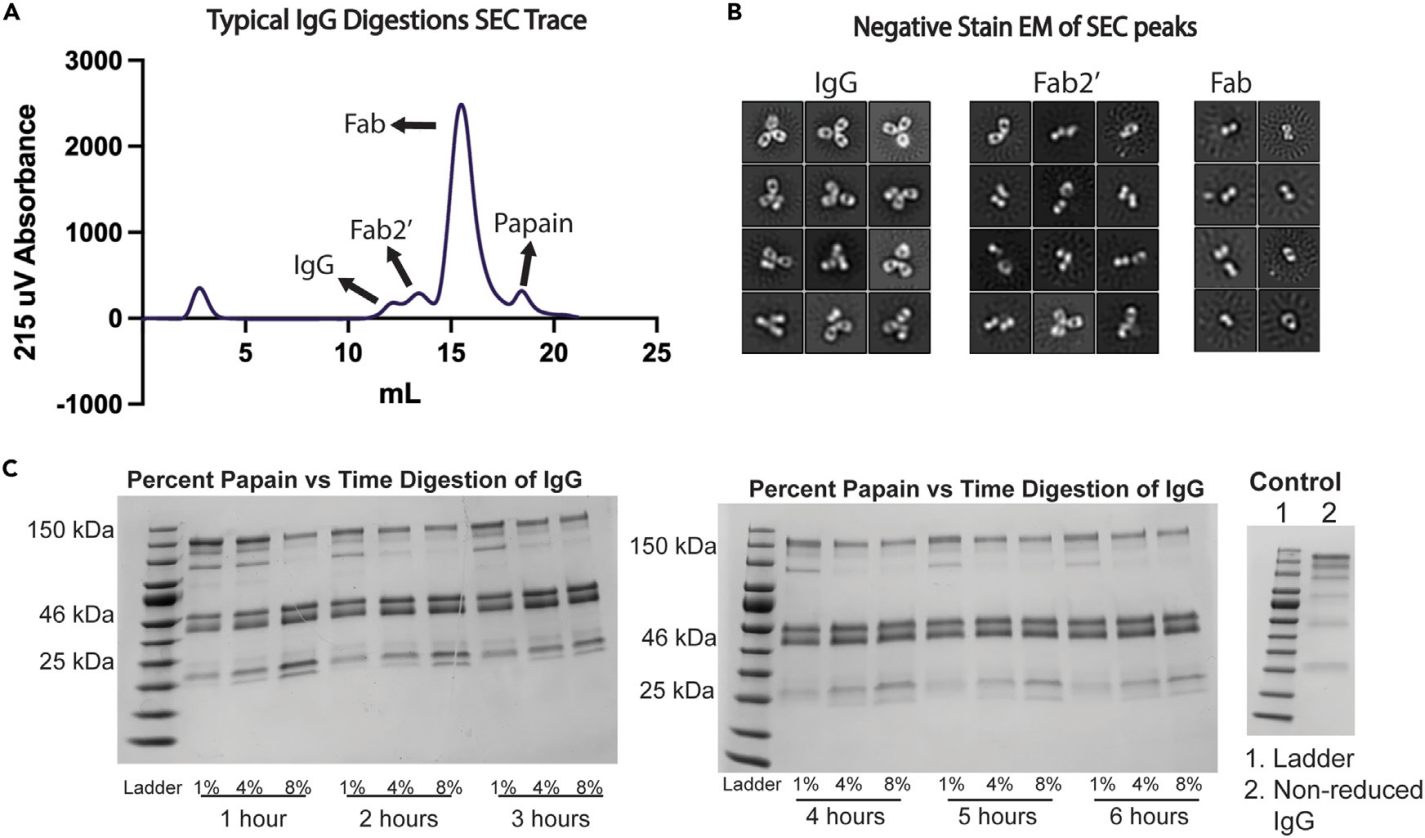
IgG Digestion with papain (A) SEC trace from S200i of digestion products. After the void peak there is an undigested IgG peak then Fab2′ shoulder with the largest peak being digested Fab followed by papain. (B) negative stain EM 2D class averages of peaks collected from SEC. (C) Percent papain versus incubation time for digestion of IgG.

After antibody digestion, we want to isolate polyclonal Fab from papain, cysteine, and iodoacetamide. Fab and Fc are similar sizes so Fc will be isolated along with Fab in SEC. The Fc region does not interfere with Fab/antigen complexes and will be included in the excess Fab peak in SEC. There is often undigested IgG which will come off before the Fab/Fc peak in SEC. Papain will come off after the Fab/Fc peak in SEC (Figure 2A) .

13. Equilibrate SEC column (we use a GE S200i) with PBS.
  ***Optional:*** Use TBS buffer instead.
14. Inject polyclonal Fab/Fc digest sample onto SEC column and collect 500 μL per well using a 96 deep-well block.
  a. Collect entire column volume.
15. Combine wells from largest peak corresponding to 50 kDa proteins (Figure 2A).
  a. Peak often has smaller peaks before and after Fab/Fc peak (Troubleshooting 1).
  b. Do not include contaminating peaks or shoulders.
16. Concentrate Fab/Fc to about 500 μL.

**Pause point:** Isolated Fab can be stored at 4°C for up to one month when 0.025% sodium azide is added, after which it is recommended to store at −80°C indefinitely.

***Note:*** Clean SEC with 1 mL 0.5 M NaOH in between sample purification runs.

**Figure 3:**
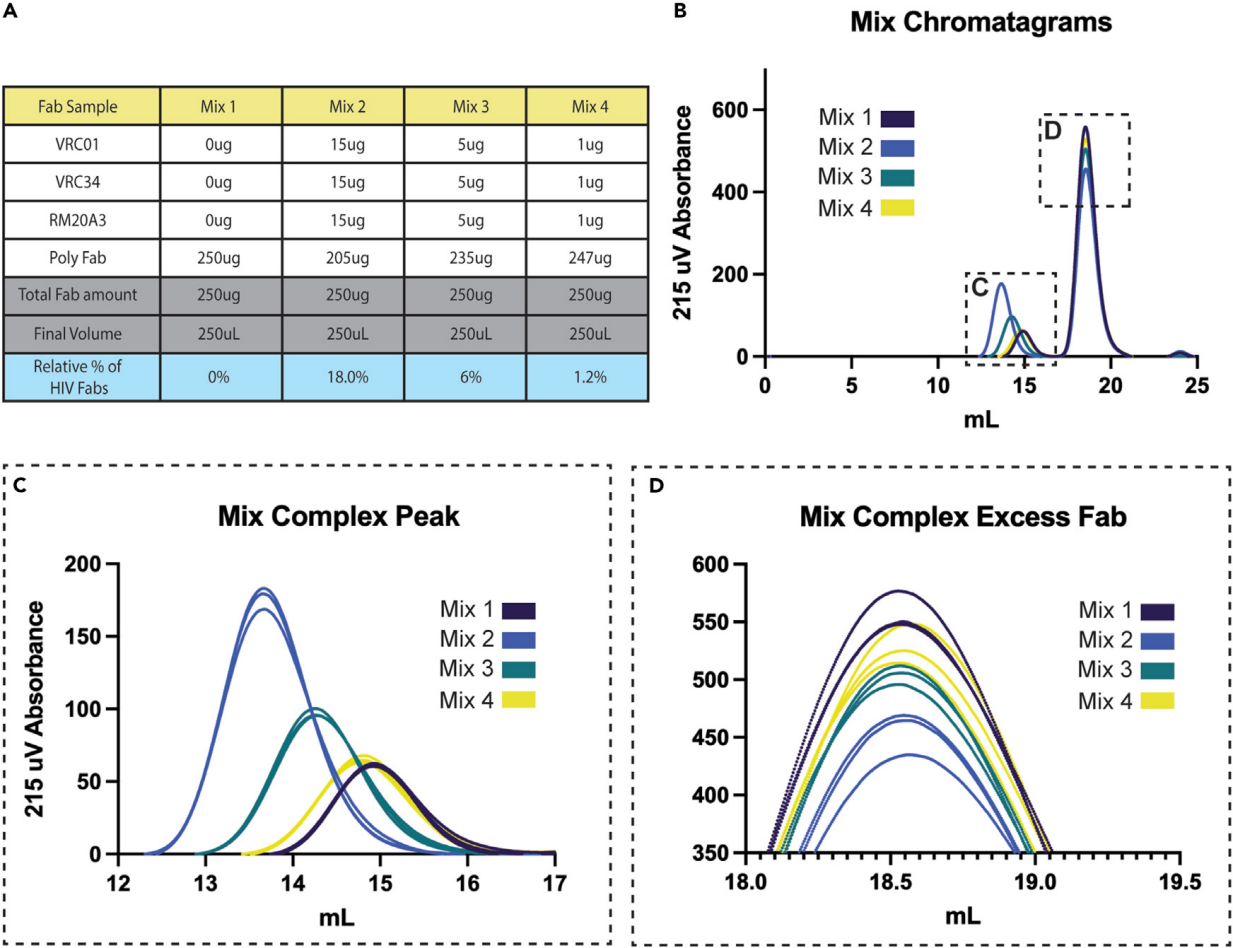
Antigen/Fab complex mixes (A) Fab combinations of Env naïve polyclonal Fab with known Env bnAbs representing possible ratios of antigen specific Fabs in polyclonal mixes added to 15 μg of. (B) SEC traces of mix combinations purified with Env focusing on complex peak. (C) SEC traces of polyclonal complex purification, zoomed in on antigen/Fab peaks. (D) Zoomed in on excess Fab peak corresponding to unbound and unspecific antigen Fabs.

### Antigen complex and purification

**Timing: 1.5 days**

An excess of polyclonal Fab (and Fc) is added to an antigen of interest. Polyclonal Fab with an affinity to the antigen will bind and the excess or non-binding Fab will elute in a separate peak in SEC. Incubating the complex overnight will give sufficient time for Fab to bind. Troubleshooting 3, 4.

17. Add 15 μg of HIV Env (antigen) to 250–500 μg of polyclonal Fab/Fc (Figure 3A).
  a. Depending on concentration of antigen-specific Fabs, more or less of polyclonal Fab/Fc can be combined.

***Note:*** 10–15 μg of antigen is enough to see an SEC peak for complex isolation at 215 nm absorbance.

***Note:*** A large molar excess of Fab is used and elutes off separately from complex so the exact ratio is less important.

18. Incubate complex overnight at 4°C.
19. Equilibrate SEC column (GE S200i) with TBS.

***Note:*** Complex needs to be purified with TBS because PBS reacts during staining and crashes out.

20. Inject complex and collect 500 μL per well using a 96 deep-well block.
  a. Collect entire column volume.
21. Combine small peak corresponding with estimated antigen/fab mass (Figures 3B and 3C).
  a. Concentrate to about 200–500 μL.

***Note:*** Concentration for negative stain EM only needs to be about 0.04 mg/mL. Dilute with TBS as needed.

22. Combine peak corresponding to excess Fab peak (Figures 3B and 3D).
  a. Save for later if needed and add 0.025% sodium azide to prevent contamination growth.

### Make negative stain EM grids

**Timing: 15 min**

Negative stain grids are made with purified Fab/antigen complexes. Troubleshooting 2.

***Note:*** Negative stain grids were made in-house. Directions as follows: 400 mesh copper grids were floated on top of a single layer of sterile collodion 2% amyl acetate, let to dry and then sputter coated with carbon using graphite rods and a Cressington carbon evaporator.

**Figure 4:**
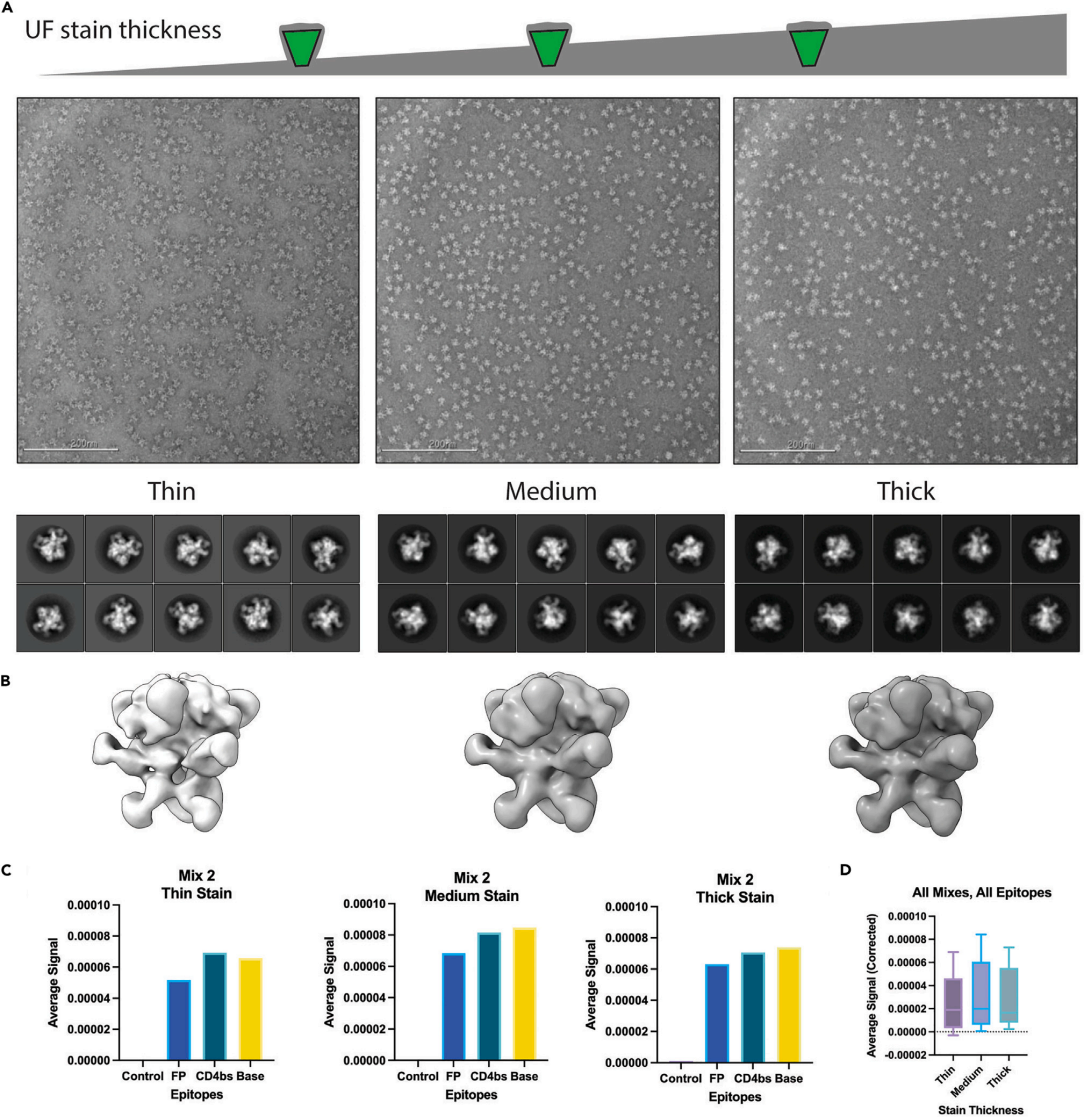
UF thickness and epitope signal (A) Cartoon representation with negative stain EM micrographs of mix 2 complexes at thin, medium, and thick UF stain thickness. 2D representative classes of mix 2 complexes. (B) 3D maps of data collected with varying UF stain thickness at ¾ volume threshold. (C) Average signal of epitope detected from data collected with varying UF stain thickness. Each dataset was processed according to steps 30–37. Average signal was calculated in Chimera by placing an 80A spherical mask over each epitope. Volume eraser tool was used to remove all external volume including antigen. The remaining volume mean with standard deviation and RMS were measured for semi-quantitative analysis. (D) Average signal of all mixes and epitopes detected of data collected with thin, medium, and thick UF stain. Scale bar is 200 nM.

***Note:*** Grids of complexes need to be made on the same day to capture representative response as Fab will disassociate overtime.

23. Although staining techniques differ between individuals, this is a good place to start.
  a. Glow discharge grid at 15 mA for 25 s.
  b. Place 2, 3 μL drops of UF onto parafilm.
  c. Load 3 μL sample at final concentration of 0.04 mg/mL onto grid.
  d. Blot with filter paper.
  e. Pick up UF droplet by blotting with grid, wait 10 s.
  f. Blot with filter paper, wait 10 s and pick up second drop of UF.
  g. Wait 50–60 s, blot with filter paper.
24. Image grid same day or screen for dilution and stain quality.
  a. See Figure 4A for ideal conditions.

**Pause point:** Stained grids are stable for months stored at 20°C.

### Collect negative stain EM data

**Timing: 2–4 h**

A negative stain grid containing polyclonal complexes from an individual timepoint is loaded onto an electron microscope and micrographs are collected for processing.

25. Collect enough micrographs for ∼100,000 initial particles (typically 200–300 micrographs).

***Note:*** We collect EMPEM data on a variety of microscopes ranging from 120 kV to 200 kV equipped with either a LaB6 or FEG electron emission source. CCD cameras that have been used are the TVIPS, Eagle and CETA. Pixel size ranges from 1.677–2.063 Ångstrom/pixel. Micrographs are .mrc files and are taken in linear mode so motion correction is not necessary. Defocus range from −1.5 to −2.5 μm. Total dose is about 25 e-/Å^2^.^4^^,^^5^ Acquisition can be automated with software such as Leginon/Appion^5^^,^^7^ or EPU (ThermoFisher).

### Processing EMPEM data

**Timing: 1–2 days**

This section describes the standard processing workflow after micrographs containing polyclonal complexes were imaged on a negative stain electron microscope.

***Note:*** The following uses RELION 3.0 software.^2^

26. Import micrographs. CTF estimation can be skipped for negative-stain data.
27. Pick particles using Laplacian-of-Gaussian autopicking and adjust minimum diameter setting. Ensure that “particles are white” is selected. If too few particles are picked, try decreasing the minimum diameter. If too much background noise is picked up, try increasing this value.
28. Extract (do not invert contrast for negatively-stained particles), and perform 2D classification (50–100 classes) using default RELION parameters.
  a. Select 2D classes that resemble antigen with or without Fabs bound. Troubleshooting 5.
29. Perform another round of 2D classification (50–100 classes).
  a. Choose 2D classes that resemble antigen.
30. Using a low pass (20 Å) filtered ligand-free antigen as the initial model, perform 3D classification (25 classes) using default RELION parameters. Troubleshooting 6.
  a. Total input particles divided by number of classes should be greater than or equal to 1000.
  b. Select 3D classes that resemble antigen with Fab bound.
31. Repeat 3D classification jobs as needed to sort maps of fab bound to antigen.
  a. Number of classes requested should follow this approximation: total input particles divided by number of classes is greater than or equal to 1000, or maximum of 40 classes.
32. Perform 3D refinements of final classes selected from 3D classification.
33. Using UCSF Chimera,^3^ isolate Fab volume from antigen with Segger tool.
  a. Save desired segment and apply Gaussian filter.
  b. Resample filtered volume on the grid of input map.
  c. Save filtered and resampled volume.
  d. The low-pass filtered initial model used in RELION can be displayed along with the segmented Fab volume to generate a composite figure.
34. Repeat for all unique epitope Fab densities.

### Expected outcomes

IgG recovery from serum will vary depending on species, concentration, and processing. Recent studies have recovered on average 8 mg of IgG from 1 mL of human serum. For 1 mL of NHP, a total of 10.5 mg was recovered although the average was roughly 6 mg. After digestion, the total recovery of Fab may drop significantly due to digestion efficiency and SEC purification. Expect to lose about half of the protein amount. 500 μg of polyclonal Fab/Fc is generally enough to see a response to the antigen, but this is heavily dependent on the study, timepoint, and relative abundance of target-specific antibodies.

Overnight incubation of antigen and polyclonal Fab should be more than enough time to see binding. Often, in sequential blood draws of a vaccination regimen, the antigen is seen to increasingly degrade with antibodies binding internal regions at later time points.^8^ Different constructs of the antigen may be needed to observe intact complexes. After purification of complex with SEC, negative stain EM grids should be made immediately because antibodies tend to fall off depending on affinity and a shift of equilibrium.

Together with serum neutralization and binding assays, e.g., enzyme linked immunosorbent assay (ELISA), this data will provide a detailed portrait of the immune response to an antigen of interest. EMPEM helps visualize antibody-mediated immune responses and allows for correlation of epitope specificities with pathogen neutralization as well as determination of antigen stability upon antigen binding. This technology is rapidly becoming an important tool in evaluating antibody responses to candidate vaccines.

## Limitations

The central limitation of this study is access and user training to electron microscopes and computational resources for data collection and processing. However, advances in both microscopy and computational technology are increasing user accessibility and decreasing costs. However, this leads to another possible limitation: cost. Electron microscopes are expensive and require service and training.

Currently, there are Electron Microscope centers funded by NIH such as NCCAT (National Center for CryoEM Access and Training), S2C2 (Stanford-SLAC Cryo-EM Center, and PNCC (Pacific Northwest Center for Cryo-EM) that offer collection services and training.

Negative stain EM provides an overview of antibody response and epitope location that can help with correlation or relationships with other immune assays. It can also identify good candidates for more in-depth investigation using cryoEMPEM as described in Antanasijevic 2022 and 2021.^9^^,^^10^ To adapt the EMEPM protocol for cryo-EM collection, a larger amount of Fab and antigen is needed. Generally, 200 μg of antigen is added to 3–7 mg of polyclonal Fab and then purified. Each cryo grid is made with 3 μL of sample at 1-2 mg/mL and freezing multiple grids of the complex is advised.

Another limitation is the nature of these samples; clinical and pre-clinical trial samples are precious in both numbers and volumes available for the number of assays researchers wish to perform. Negative stain EMPEM can be done with as little as 150–250 μg of Fab. However, this requires 1 mL–2 mL of starting plasma or sera. Additionally, handling of sera and plasma requires proper PPE, safety protocols and training.

Finally, differences in affinity, abundance, and epitope overlap of polyclonal Fabs will affect which antibodies will be recovered during image processing. Antibodies targeting the same epitope may out-compete other antibodies or occlude nearby epitopes from being targeted. Highly potent antibodies with low abundance or high off rates may remain undetectable.

## Troubleshooting

### Problem 1: Incomplete IgG digestion

(Related to step 11) IgG peaks are visible on SEC trace during Fab/Fc purification (“Fab/Fc purification”, 16) Figure 2A is a “normal” or typically observed result from digesting with 4% papain w/w for 4 h. Different types of IgG from various sources may need to be digested for a longer amount of time or incubate with a higher concentration of papain (Figure 2C). It is common that papain is unable to digest some percentage of IgG.

### Potential solution

Collect wells from IgG peak and screen in negative stain EM (Figure 2B) to confirm peak is undigested IgG and not contamination. Then perform test digestions to determine if other parameters will work such as increased time or papain percent (Figure 2C).

• Test digest 25 μg of polyclonal IgG for a total reaction of 50 mL.
  ○ Remove 9 μL digest at each timepoint and add 1 μL iodoacetamide to stop reaction.
  ○ Run a non-reduced SDS-PAGE gel of time points along with undigested IgG.

### Problem 2: Negative stain is uneven on grid

(Related to step 23) Although collecting data with medium stain is ideal, collecting on a variety of stain thicknesses will still allow specific epitopes to be identified (Figure 4). Some epitopes may be at low occupancy and collecting more data is helpful to find them.

### Potential solution

• Screen negative stain EM grids for ideal UF thickness and particle density.
• Individual particles should not be touching for proper picking.

### Problem 3: Inability to separate epitopes

(Related to step 17) If antigen is completely occupied by Fabs, it may be difficult to isolate individual epitopes.

### Potential solution

Make another complex with half as much Fab. Can also try taking excess fab from first round and performing another round of complex purification.

### Problem 4: Low binding occupancy or high off rate

(Related to step 17).

### Potential solution

Try cross-linking Fabs to antigen. See Torrents de la Peña 2023^11^ for detailed discussion and method.

### Problem 5: Antigen degradation

(Related to step 28) As described in Turner 2021,^8^ antibody mediated trimer disassembly is seen to increase in later timepoints during vaccine trials. This will be visible during 2D classification.

### Potential solution

• Perform EMPEM on an earlier timepoints for vaccine trials. The Fab that causes disassembly might be at lower concenetration or less potent at earlier timepoints.
• Try more stabilized antigens if possible. Can also try crosslinking the trimer before adding polyclonal Fab.
• Incubate with multiple constructs of antigen as done in Bangaru 2022.^12^

### Problem 6: Orientation bias in 2D and 3D classification

(Related to step 30).

### Potential solution

Add a fiducial monoclonal Fab to help with tumbling on negative stain grid. Incubate with high affinity, fiducial Fab for 2 h before adding polyclonal fab and incubating overnight.

## Resource availability

### Lead contact

Further information and requests for resources and reagents should be directed to and will be fulfilled by the lead contact, Andrew Ward, andrew@scripps.edu.

### Materials availability

This study did not generate new unique reagents.

### Data and code availability

This study did not generate or analyze unique datasets or code. However, most EMPEM studies will generate electron microscopy datasets which should be deposited in an appropriate repository such as the Electron Microscopy Databank (EMDB) or the Protein Data Bank (PDB).
